# Affective temperaments, self-schemas, and their interplay with emotional distress

**DOI:** 10.3389/fpsyt.2025.1732425

**Published:** 2026-01-15

**Authors:** Manel Monsonet, Karen Fagian-Núñez, Thomas R. Kwapil, Neus Barrantes-Vidal

**Affiliations:** 1 Departament de Psicologia Clínica i de la Salut, Universitat Autònoma de Barcelona, Barcelona, Spain; 2 Department of Psychology, University of Illinois at Urbana-Champaign, Urbana-Champaign, Ill, United States; 3 Centre for Biomedical Research Network on Mental Health (CIBERSAM), Instituto de Salud Carlos III, Madrid, Spain

**Keywords:** affective temperaments, anxiety symptoms, depressive symptoms, mediation analyses, self-schemas, temperament

## Abstract

**Background:**

Affective temperaments and self-schemas are theorized to shape susceptibility to emotional distress, yet their interplay remains empirically unexplored. This study investigates (1) associations between affective temperaments and positive and negative self-schemas, and (2) whether positive and negative self-schemas mediate the temperament-distress relationship.

**Methods:**

A cross-sectional sample of 808 young adults (mean age = 20.8; 77.2% female) completed the TEMPS-A (affective temperaments), BCSS (self-schemas), and SCL-90-R (depressive/anxiety symptoms). Spearman correlations and parallel mediation analyses tested hypotheses.

**Results:**

Positive self-schemas showed a positive association with hyperthymic temperament and inverse association with cyclothymic and depressive temperaments. Negative self-schemas were associated with cyclothymic and depressive temperaments and inversely associated with hyperthymic temperament. Mediation analyses revealed that both positive and negative self-schemas significantly mediated the relationships between cyclothymic, depressive, hyperthymic, and anxious temperaments with depressive symptoms. Conversely, positive self-schemas only mediated the pathway from hyperthymic temperament to anxiety symptoms, whereas negative self-schemas mediated all temperament-anxiety pathways.

**Conclusion:**

This study provides the first empirical evidence that self-schemas act as mediating mechanisms linking affective temperaments to affective symptoms. Our findings thereby support a biopsychosocial model of emotional distress, founded on the interaction between genetically-influenced temperaments and socially-constructed self-schemas. Consequently, therapeutic interventions designed to modify self-schemas may represent an effective strategy for mitigating the pathway from temperamental vulnerability to emotional distress.

## 1 Introduction

Inherent individual characteristics and traits related to emotional distress and mood patterns have played a significant role in the study of human behavior and health since ancient times. The theory of humors was established by the Greek philosophers Hippocrates and his pupil Polybos, although it may have been present in ancient Mesopotamia (1). Rooted in this theory, four temperamental types were described and later developed by Galen, who refers to temperaments as bodily and psychosocial dispositions that influence emotional and behavioral inclinations and the susceptibility to certain diseases (2).

More recently, Akiskal and colleagues developed a theory of affective temperaments (3–5), which emerged from clinical observations and theorical considerations based partly on previous seminal works of Kraepelin (6), Kretschmer (7) and Schneider (8). In the latest version of this theory, five affective temperament types were included: depressive, cyclothymic, irritable, hyperthymic, and anxious. These temperaments, which are present as widespread dimensions in the general population (9), attempt to capture trait affective dispositions to mood disorders, but they may also have an adaptative role (10, 11). Briefly defined, depressive temperament encompasses pessimistic thinking, doubtfulness, sadness, and dissatisfaction; cyclothymic temperament displays sudden shifts in mood and energy, and instability in self-concept, socialization, and sleep patterns; irritable temperament is characterized by annoyance, anger, and propensity for aggression; hyperthymic temperament includes optimism, extraversion, high energy, but also risk-taking and overconfidence; and anxious temperament encompass fearfulness and excessive apprehension.

Previous research in clinical samples has shown the relevance of affective temperaments in the development and course of mood disorders (12, 13), as well as their utility to distinguishing between unipolar from bipolar disorders and between bipolar disorders subtypes (14). Affective temperaments have also been related to suicidal behavior (15), treatment adherence (16), personality disorders (17), and anxiety disorders (18). In nonclinical samples mounting evidence has found a clear association between affective temperaments and depressive and anxiety symptoms. Depressive, cyclothymic, irritable and anxious temperaments have been consistently correlated with higher levels of anxiety and depressive symptoms (19–22). Regarding hyperthymic temperament, negative correlations with anxiety and depressive symptoms have been reported (20, 23, 24), as well as positive associations with hypomania, cyclothymia, substance abuse and impulse control disorders (24–26). DeGeorge and colleagues (27) reported that cyclothymic, irritable, and hyperthymic temperaments predicted the development of mood disorders in a three-years longitudinal study.

Although temperament is considered to have a strong genetic basis and is therefore highly heritable (28), core schemas are thought to develop through early experiences and social interactions (29)—even though these are not independent constructs as individual differences in temperament will have some impact on the appraisal and response to experiences (30). Core schemas, or core beliefs, are foundational concepts within cognitive-behavioral theory that serve as fundamental templates for organizing one’s perception of reality (31). They are defined as deeply held, pervasive, and enduring cognitive structures that have a central role in the appraisal of experience (32). Schemas are formed from repeated patterns of experience, particularly in childhood, and become automated, often operating outside conscious awareness. They refer to an individual’s appraisals of themselves, other people, and the world around, and persist as relatively stable constructs. A core tenet of schema theory is that once activated, a schema biases information processing in a schema-consistent manner through processes of cognitive distortion, such as selective attention and overgeneralization (33). Core schemas have been widely studied in relation to psychopathology (34–36) and have been described as important mechanisms in the development of affective disorders (37–39). Moreover, they have historically been a key component in cognitive theories of mood and anxiety disorders (31, 40).

Within core schemas, self-schemas can be defined as cognitive generalizations specifically concerning the self. They are the integrated set of memories, beliefs, and generalizations that constitute an individual’s self-concept (41, 42). Self-schemas can be characterized by their valence: negative versus positive. While negative self-schemas encapsulate the maladaptive, self-referential content of dysfunctional core beliefs (e.g., “I am worthless,” “I am a failure”), positive self-schemas reflect stable, positive, and resilient beliefs about the self (e.g., “I am capable,” “I am resilient”). Cognitive theories of anxiety, depressive, personality and psychotic disorders have included negative self-schemas (or maladaptive schemas) to deepen the understanding of the causes of mental health problems or difficulties as well as to design more personalized interventions (33). Accordingly, extensive research has focused on studying the detrimental impact of negative self-schemas on the development and exacerbation of such disorders (43–46). However, positive self-schemas may act as a buffering factor for psychopathology and a lack of positive self-schemas may contribute to the causal and developmental factors of mental health problems (47, 48). Although evidence suggests that positive self-schemas are not at the opposite end of negative self-schemas and exist in a separate continuum (49), only a paucity of studies have explored the impact of positive self-schemas on psychological disorders (50–52).

It has been pointed out that self-evaluations, particularly negative self-schemas, are associated with dysfunctional emotional responses (53), and that self-schemas may be a central mediating factor that may account for the relationship between stressful life events and psychopathology (41). However, only a paucity of studies has investigated the mediating role of maladaptive schemas in the pathway from temperament to psychopathology (54–56). Furthermore, to our knowledge, no studies have investigated how affective temperaments are associated with self-schemas, or whether self-schemas could account for the link between affective temperaments and emotional distress.

**Figure 1:**
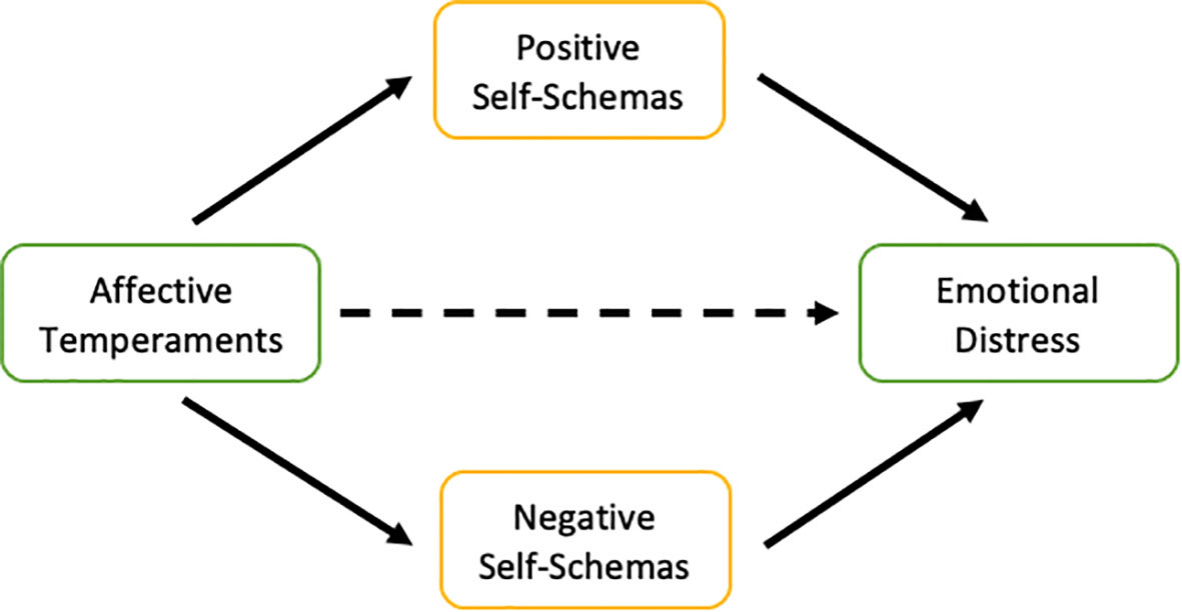
Conceptual diagram of the mediation models tested in the present study.

The primary aim of this study was to explore, in a large sample of young adults, the associations of affective temperaments and self-schemas with emotional distress. Emotional distress is commonly defined as a broad affective state characterized by heightened negative emotionality and subjective psychological discomfort, typically manifesting through symptoms of depression and anxiety (e.g., 57–59). Consistent with this conceptualization, we operationalized emotional distress using validated measures of depressive and anxiety symptoms. First, we examined the associations between affective temperaments and self-schemas. Consistent with theorical frameworks reviewed above, we hypothesized that cyclothymic, depressive, and anxious temperaments will be associated with higher levels of negative self-schemas, whereas hyperthymic temperament will be associated with greater levels of positive self-schemas. Second, we examined whether positive and negative self-schemas simultaneously mediate the relationship between affective temperaments and depressive/anxiety symptoms (see **Figure 1**). Given that this objective remains empirically untested, it was exploratory in nature. Nevertheless, guided by conceptual frameworks, we hypothesized that both positive and negative self-schemas would account for the relationship between affective temperaments—specifically depressive, cyclothymic, and hyperthymic temperaments—and depressive/anxiety symptoms.

## 2 Method

### 2.1 Design, participants, and procedure

This cross-sectional study analyzed data from the first wave (T1) of the Barcelona Longitudinal Investigation of Schizotypy-I (BLISS-I), a multi-wave study designed to investigate risk factors for psychopathology (60, 61). Participants were 808 young adults from the Barcelona area, Spain. The sample comprised two subgroups: 547 students enrolled in psychology courses at the Universitat Autònoma de Barcelona (UAB) and 261 individuals recruited from local technical schools. Participants were recruited through institutional announcements and participated voluntarily. Inclusion criteria for the parent BLISS-I study was sufficient fluency in Spanish to complete the questionnaires. Exclusion criteria for the present analysis were: (1) presence of invalid self-report measures, and (2) failure to provide informed consent. No participants were excluded based on clinical status, ensuring an unselected community sample. After providing informed consent, participants completed a battery of self-report questionnaires. The study was approved by the Universitat Autònoma de Barcelona Ethics Committee (CEEAH: 701H-JS).

### 2.2 Materials

#### 2.2.1 Anxious and affective temperaments

The Temperament Evaluation of Memphis, Pisa, Paris, and San Diego Autoquestionnaire (TEMPS-A) (11) is a widely used self-report questionnaire composed of five subscales that assess anxious, cyclothymic, irritable, hyperthymic, and dysthymic temperaments. These subscales are designed to capture not only emotional and cognitive patterns of behavior but also psychomotor and circadian rhythms that may predispose individuals to mood disorders (62). For the present study the short version (63) consisting of 39 true/false items was used. Higher scores indicate greater levels of the respective temperament.

#### 2.2.2 Self-schemas

The Brief Core Schema Scales (BCSS) (41) is a self-administered questionnaire designed to capture beliefs concerning oneself and others. It consists of four subscales (positive-self, negative-self, positive-others, and negative-others), each comprised of six statements rated on a five-point Likert scale (0 to 4), with lower scores reflecting lower levels of the specific schemas. In the current study, only the positive-self and negative-self subscales were considered, as the theoretical focus is on self-referential cognitive processes, which are more directly implicated in cognitive theories of depression and anxiety (43).

#### 2.2.3 Anxiety and depression

Symptoms of depression and anxiety were assessed using the respective subscales of the Spanish version of the Symptom Checklist-90-Revised (SCL-90-R) (64, 65). The depression subscale contains 13 items, whereas the anxiety subscale consists of 10 items. Each item is rated on a five-point scale (0 = ‘not at all’ to 4 = ‘extremely’), with higher scores indicating greater symptom severity.

### 2.3 Data analysis

Statistical analyses were conducted using Jamovi v2.3 (66), which operates within the R statistical environment version 4.1 (67). Correlational analyses were conducted using Spearman’s rho (*ρ*) to assess relationships between self-schemas and affective temperaments. Shapiro-Wilk tests confirmed all variables significantly deviated from normality (*W* = 0.809–0.990, *ps* <.001) and density plots revealed right-skewed distributions, supporting the use of non-parametric analyses. Given the multiple comparisons problem inherent to correlation matrices (21 pairwise tests in this study), a Bonferroni correction was applied to control the familywise error rate (FWER). The adjusted significance threshold was set at *p* <.0024 (0.05/21 tests), ensuring ≤5% probability of any false positives across all results.

Parallel mediation analyses were conducted to investigate whether positive and negative self-schemas mediated the relationships between affective temperaments and emotional symptom outcomes. The independent variables were the five affective temperaments (cyclothymic, depressive, hyperthymic, irritable, anxious), and the dependent variables were depressive symptoms and anxiety symptoms. Both positive and negative self-schemas were entered as simultaneous mediators in each model. Each model examined the pathway from one affective temperament to one symptom outcome, assessing the unique indirect effects of both self-schema types while controlling for their shared variance. A parallel mediation model was chosen because it allowed us to examine the unique indirect effect of each mediator (positive and negative self-schemas) while statistically controlling for the other. All mediation models included age and gender as covariates. The jAMM module (68) was employed, which implements structural equation modeling through the lavaan package (69). Bias-corrected 95% confidence intervals for these effects were derived from 5,000 bootstrap resamples, with effects considered statistically significant when the CIs excluded zero. The proportion of mediated effect was calculated for significant pathways to quantify each mediator’s contribution.

Although the study was not pre-registered, it was limited to the specified analyses assessing the associations of temperaments, self-schemas, and symptoms. Furthermore, the results of all analyses are reported.

## 3 Results

The final sample consisted of 799 participants. The mean age was 20.8 years (SD = 4.06) and the majority of participants were female (77.2%). Descriptive statistics for all study variables are provided in **Table 1**.

**Table 1:** Descriptive statistics for study variables and Spearman correlations between self-schemas, affective temperaments, and depressive and anxiety symptoms.

Variable	Mean (SD)	Observed Range	1	2	3	4	5	6	7	8
1. Positive Self-Schemas	13.36 (4.59)	0-24	--							
2. Negative Self-schemas	2.25 (2.53)	0-16	-0.33^*^	--						
3. Cyclothymic Temperament	4.16 (2.70)	0-12	-0.25^*^	0.35^*^	--					
4. Depressive Temperament	2.25 (1.72)	0-8	-0.36^*^	0.41^*^	0.38^*^	--				
5. Hyperthymic Temperament	4.06 (2.04)	0-8	0.40^*^	-0.21^*^	-0.06	-0.21^*^	--			
6. Irritable Temperament	2.20 (1.59)	0-8	-0.06	0.07	0.28^*^	0.23^*^	0.06	--		
7. Anxious Temperament	1.16 (1.05)	0-3	-0.09^*^	0.13^*^	0.30^*^	0.17^*^	0.01	0.17^*^		
8. Depressive Symptoms	11.60 (8.60)	0-49	-0.27^*^	**0.41** ^*^	*0.57* ^*^	0.43^*^	-0.16^*^	0.22^*^	0.26^*^	--
9. Anxiety Symptoms	6.28 (5.58)	0-33	-0.16^*^	0.28^*^	0.45^*^	0.31^*^	-0.02	0.27^*^	0.29^*^	***0.62*** ^*^

*Significant correlations after Bonferroni correction (*p*  < .0024).

SD, Standard Deviation. Medium effect sizes in bold, large effect sizes in bold and italics.

### 3.1 Correlations of affective temperaments with positive and negative self-schemas

Spearman correlation analyses revealed significant relationships between both positive and negative self-schemas and affective temperaments (see **Table 1**). Positive self-schemas were moderately and inversely associated with negative self-schemas, supporting that they are not merely the opposite end of a single dimension. As expected, positive self-schemas showed a moderate positive correlation with hyperthymic temperament, and negative correlations of weak and moderate strength with cyclothymic and depressive temperaments, respectively. In contrast, negative self-schemas were moderately associated with cyclothymic and depressive temperaments but exhibited a weak negative correlation with hyperthymic temperament. Finally, a small yet significant positive correlation emerged between anxious temperament and negative self-schemas. Other correlations between self-schemas and temperaments were statistically non-significant.

### 3.2 Intercorrelations among affective temperaments

Regarding associations between temperaments, only two moderate correlations emerged: cyclothymic temperament with both depressive and anxious temperaments. Cyclothymic temperament also showed a significant but weaker association with irritable temperament, whereas the link between depressive and irritable temperaments was similarly weak. In contrast, hyperthymic temperament exhibited only a weak inverse correlation with depressive temperament. Other correlations among affective temperaments were either statistically non-significant or of minimal magnitude.

### 3.3 Mediation effects of affective temperaments on emotional symptoms via self-schemas

Parallel mediation analyses for depressive symptoms are shown in **Table 2**. Negative self-schemas mediated all the pathways between affective temperaments and depressive symptoms, but their influence was particularly pronounced for depressive (b = 0.647, proportion mediated = 0.26) and hyperthymic (b = -0.376, proportion mediated = 0.64) temperaments. There was a significant positive indirect effect, indicating that higher levels of depressive, cyclothymic, irritable, and anxious temperaments predicted higher depressive symptoms via their association with higher levels of negative self-schemas. Conversely, there was a significant negative indirect effect, indicating that higher levels of hyperthymic temperament predicted lower depressive symptoms via its association with lower levels of negative self-schemas. Negative self-schemas consistently demonstrated stronger mediation effects than positive self-schemas, accounting for 17-64% of associations.

**Table 2:** Mediation analyses examining the indirect effects of temperaments on depressive symptoms via positive and negative self-schemas.

Effects	Raw parameter estimate	S.E.	95% C.I.		β	Proportion mediated
			Lower	Upper		
Cyclothymic temperament on depressive symptoms						
Total effect	1.873^**^	0.091	1.678	2.112	0.589	
Direct effect	1.500^**^	0.093	1.291	1.689	0.472	
Indirect effect via positive self-schemas	0.064^**^	0.025	0.022	0.118	0.020	0.03
Indirect effect via negative self-schemas	0.309^**^	0.045	0.210	0.438	0.097	0.17
Total indirect effect	0.373^**^	0.052	0.272	0.474	0.117	0.20
Hyperthymic temperament on depressive symptoms						
Total effect	-0.585^**^	0.148	-0.900	-0.289	-0.139	
Direct effect	-0.065	0.144	-0.214	0.339	0.453	
Indirect effect via positive self-schemas	-0.274^**^	0.065	-0.413	-0.157	-0.065	0.47
Indirect effect via negative self-schemas	-0.376^**^	0.068	-0.561	-0.234	-0.089	0.64
Total indirect effect	-0.650^**^	0.094	-0.834	-0.466	-0.154	1.11
Depressive temperament on depressive symptoms						
Total effect	2.447^**^	0.186	2.085	2.818	0.491	
Direct effect	1.676^**^	0.167	1.300	2.084	0.336	
Indirect effect via positive self-schemas	0.123^*^	0.059	0.014	0.243	0.025	0.05
Indirect effect via negative self-schemas	0.647^**^	0.088	0.450	0.893	0.130	0.26
Total indirect effect	0.770^**^	0.106	0.562	0.978	0.155	0.31
Irritable temperament on depressive symptoms						
Total effect	1.211^**^	0.186	0.807	1.638	0.223	
Direct effect	0.961^**^	0.164	0.608	1.336	0.177	
Indirect effect via positive self-schemas	0.044	0.029	-0.007	0.116	0.008	0.04
Indirect effect via negative self-schemas	0.205^**^	0.078	0.047	0.404	0.038	0.17
Total indirect effect	0.249^*^	0.083	0.086	0.412	0.046	0.21
Anxious temperament on depressive symptoms						
Total effect	1.885^**^	0.282	1.316	2.469	0.230	
Direct effect	1.438^**^	0.249	0.938	1.946	0.176	
Indirect effect via positive self-schemas	0.091^*^	0.046	0.009	0.210	0.011	0.05
Indirect effect via negative self-schemas	0.356^**^	0.119	0.135	0.642	0.044	0.19
Total indirect effect	0.447^**^	0.128	0.197	0.697	0.055	0.24

S.E., standard error; C.I., Bias-corrected 95% confidence intervals derived from 5,000 bootstrap resamples. Proportion mediated values greater than 1.0 indicate that the indirect effect exceeds the total effect in magnitude, suggesting complete mediation.

*p<.05; **p<.01.

Positive self-schemas significantly mediated the relationship between affective temperaments and depressive symptoms for all temperaments except the irritable temperament. There was a significant positive indirect effect, indicating that higher levels of depressive, cyclothymic, and anxious temperaments predicted higher depressive symptoms via their association with lower levels of positive self-schemas. Conversely, there was a significant negative indirect effect, indicating that higher levels of hyperthymic temperament predicted lower depressive symptoms via its association with higher levels of positive self-schemas. For irritable temperament, the mediating effect of positive self-schemas was not significant when the model accounted for the shared variance with negative self-schemas. The influence of positive self-schemas was relatively modest, accounting for 3-5% of mediation in most temperaments. However, positive self-schemas played a more substantial role in the association between hyperthymic temperament and depressive symptoms (b = -0.274, proportion mediated = 0.47).

**Table 3** presents the results of parallel mediation analyses for anxiety symptoms. Negative self-schemas consistently mediated anxiety symptoms across all temperaments with the strongest mediation observed for depressive (b = 0.321, proportion mediated = 0.3) and hyperthymic (b = -0.185, proportion mediated = 3.78) temperaments. Specifically, there was a significant positive indirect effect, whereby higher levels of depressive, cyclothymic, irritable, and anxious temperaments predicted greater anxiety symptoms through increased negative self-schemas. Conversely, a significant negative indirect effect indicated that a higher hyperthymic temperament predicted lower anxiety symptoms via its association with reduced negative self-schemas. The magnitude of this indirect effects relative to the near-zero total effect resulted in large proportion mediated value (3.78), indicating that the relationship operates almost entirely through self-schema pathways.

**Table 3:** Mediation analyses examining the indirect effects of temperaments on anxiety symptoms via positive and negative self-schemas.

Effects	Raw parameter estimate	S.E.	95% C.I.		β	Proportion mediated
			Lower	Upper		
Cyclothymic temperament on anxiety						
Total effect	0.967^**^	0.065	0.819	1.114	0.468	
Direct effect	0.823^**^	0.069	0.670	0.978	0.398	
Indirect effect via positive self-schemas	0.009	0.017	-0.022	0.046	0.004	0.01
Indirect effect via negative self-schemas	0.135^**^	0.029	0.070	0.210	0.065	0.14
Total indirect effect	0.144^*^	0.034	0.078	0.210	0.069	0.15
Hyperthymic temperament on anxiety						
Total effect	-0.049	0.098	-0.244	0.149	0.018	
Direct effect	0.261^*^	0.101	0.053	0.462	0.095	
Indirect effect via positive self-schemas	-0.124^**^	0.045	-0.215	-0.039	0.045	2.53
Indirect effect via negative self-schemas	-0.185^**^	0.036	-0.288	-0.110	0.068	3.78
Total indirect effect	-0.309^**^	0.058	-0.422	-0.196	0.113	6.31
Depressive temperament on anxiety						
Total effect	1.083^**^	0.108	0.843	1.324	0.334	
Direct effect	0.743^**^	0.122	0.480	1.004	0.229	
Indirect effect via positive self-schemas	0.020	0.043	-0.059	0.107	0.006	0.02
Indirect effect via negative self-schemas	0.321^**^	0.059	0.186	0.479	0.099	0.30
Total indirect effect	0.341^**^	0.073	0.198	0.484	0.105	0.32
Irritable temperament on anxiety						
Total effect	1.004^**^	0.119	0.739	1.278	0.286	
Direct effect	0.902^**^	0.113	0.658	1.157	0.257	
Indirect effect via positive self-schemas	0.012	0.010	-0.002	0.044	0.003	0.01
Indirect effect via negative self-schemas	0.090^*^	0.036	0.018	0.198	0.026	0.09
Total indirect effect	0.102^*^	0.037	0.029	0.175	0.029	0.10
Anxious temperament on anxiety						
Total effect	1.639^**^	0.180	1.257	2.027	0.309	
Direct effect	1.451^**^	0.172	1.092	1.815	0.274	
Indirect effect via positive self-schemas	0.025	0.018	-0.009	0.080	0.005	0.02
Indirect effect via negative self-schemas	0.163^**^	0.056	0.058	0.306	0.031	0.10
Total indirect effect	0.188^**^	0.059	0.073	0.303	0.036	0.12

S.E., standard error; C.I., Bias-corrected 95% confidence intervals derived from 5,000 bootstrap resamples. Proportion mediated values greater than 1.0 indicate that the indirect effect exceeds the total effect in magnitude, suggesting complete mediation.

*p<.05; **p<.01.

Positive self-schemas demonstrated a significant mediating effect for the relationship between hyperthymic temperament and anxiety symptoms, accounting for a substantial mediation effect that exceeds the magnitude of the total effect (b = -0.124, proportion mediated = 2.53). This indicates that higher levels of hyperthymic temperament predicted lower anxiety symptoms through its association with higher levels of positive self-schemas. When modeled concurrently with negative self-schemas, no other indirect effects through positive self-schemas remained significant.

## 4 Discussion

This study sought to examine the associations of affective temperaments and self-schemas, and their interplay in relation to anxiety and depressive symptoms. To our knowledge, no prior studies have investigated self-schemas as a mediator between affective temperaments and affective distress. Our findings indicated a coherent pattern of associations among self-schemas and affective temperaments, showing that positive self-schemas are correlated with hyperthymic temperament, while showing an inverse association with cyclothymic and depressive temperaments. Conversely, negative self-schemas were inversely related to hyperthymic temperament but positively associated with cyclothymic and depressive temperaments. Our mediation analyses revealed that both positive and negative self-schemas mediated the associations between affective temperaments (cyclothymic, depressive, hyperthymic, and anxious) and depressive symptoms. These findings seem to demonstrate that both positive and negative self-schemas account for depressive symptoms across most affective temperaments. Negative self-schemas also mediated anxiety symptoms across all temperaments, whereas positive self-schemas exerted a buffering effect only for anxiety associated with hyperthymic temperament. In summary, this study detailed the associations of self-schemas with specific affective temperaments, highlighting self-schemas as a key mechanism linking affective temperaments to emotional symptoms. This underscore self-schemas as potential modifiable therapeutic targets that may mitigate the association of temperament and affective distress. Focusing on these more malleable cognitive structures could thereby alleviate emotional symptoms, even for individuals with a vulnerable affective temperament.

### 4.1 Associations between affective temperaments and self-schemas

As hypothesized, positive self-schemas demonstrated the strongest relationship with hyperthymic temperament, consistent with theorical conceptions of this temperament as characterized by cheerfulness, over-confidence, and positive affectivity (5). These findings align with research linking specific positive schema themes—such as self-efficacy, optimism, and worthiness—to psychological well-being and resilience (70), suggesting that hyperthymic temperament may exert a consistent influence on cognitive self-representations. The negative association between positive self-schemas with depressive and cyclothymic temperaments is line with prior evidence supporting the inverse relationship between positive self-schemas and depressive symptoms (50), suggesting a protective role of positive self-schemas against mood-related vulnerabilities (71). In contrast, positive self-schemas appeared relatively independent of irritable and anxious temperaments, indicating a certain specificity in their relationships with affective traits. Nevertheless, further research is needed to replicate and confirm these findings.

Consistent associations emerged for negative self-schemas with both cyclothymic and depressive temperaments. Theoretical models propose that these temperaments are characterized by emotional instability, negative self-referential thinking, and proneness to depressive symptoms (10, 72),, which may reinforce and perpetuate dysfunctional schemas over time. Negative self-schemas were inversely associated with hyperthymic temperament, suggesting that hyperthymic temperament may buffer against adverse self-representations. Conversely, low levels of traits such as optimism or vitality characterizing the hyperthymic temperament could limit psychological resources like adaptative coping styles, thus exacerbating vulnerability to self-negative views (4, 73). Finally, correlations between negative self-schemas and irritable and anxious temperaments were either non-significant or negligible, a pattern that diverges from our initial hypotheses. This suggest that negative self-schemas are less central to anxiety, and especially, irritable temperaments; nevertheless, further studies are needed to explore the possibility of more subtle or context-dependent associations in samples with more severe temperamental manifestations and using methodologies that account for potential moderators like stress exposure.

### 4.2 Self-schemas as mediators between affective temperaments and depressive-anxiety symptoms

Self-schemas mediated the relationship between affective temperaments and emotional symptoms, with indirect effects being more pronounced for depressive than for anxiety symptoms. Negative self-schemas consistently emerged as a robust mediator across most temperaments, whereas positive self-schemas played a comparatively weaker role—significantly influencing depressive symptoms in most cases but showing no significant mediation for anxiety symptoms (except in hyperthymic temperament).

The indirect effects via negative self-schemas accounted for a substantial proportion of the total effects. Notably, depressive temperament – characterized by pessimism and low self-worth- exhibited a robust mediation through negative self-schemas (26% for depressive symptoms, 30% for anxiety symptoms). Similarly, cyclothymic temperament, which involves mood instability and hyper-reactivity, demonstrated a significant indirect effect, suggesting that emotional dysregulation in these temperaments may be exacerbated by negative self-perceptions, reinforcing a cycle of distress. Negative self-schemas also accounted for the indirect effects of irritable and anxious temperament on both depressive and anxiety symptoms. Together, these findings support the idea that temperamental predispositions and negative self-schemas interact to heighten psychopathology risk and also seem to reinforce the transdiagnostic role of negative self-schemas. This aligns with cognitive theories positing that negative self-schemas serve as a core mechanism through which affective temperaments predispose individuals to emotional distress (32, 74).

The mediating role of positive self-schemas in depressive symptoms was modest, with effects consistently smaller than those of negative self-schemas. This suggests that while positive self-schemas may provide some buffering effect, their hypothesized protective influence seems overshadowed by the stronger contribution of negative self-schemas in the pathway to depressive symptoms. The contrast was even more pronounced in the pathways to anxiety symptoms, where analyses revealed null mediation effects of positive self-schemas across cyclothymic, depressive, irritable, and anxious temperaments once negative self-schemas were accounted for. These findings underscore that positive self-schema seem to play a secondary role in temperament-emotional symptoms pathways when modeled concurrently with negative self-schemas. Their limited effects suggest that positive self-schemas, though potentially protective, seems not sufficient to counterbalance the joint influence of temperament and negative self-cognitions. Further research is needed to replicate these findings and examine their generalizability to clinical populations. Similarly, this finding should be interpreted with caution in the context of adult populations, given that the integration of stable positive self-views is a known developmental challenge in younger individuals (75).

Finally, the findings regarding hyperthymic temperament warrant separate discussion, as self-schemas demonstrated stronger mediation effects. The non-significant direct effect between hyperthymic temperament and depressive symptoms suggest that its impact operates almost entirely through positive and negative self-schemas. However, a high hyperthymic temperament may still predispose individuals to bipolar disorders (24, 76). A similar pattern, though attenuated, emerged for anxiety symptoms. Notably, the combined mediation via both self-schema types underscores their importance as a key mechanism linking hyperthymic temperament to anxiety symptomatology. Unlike other temperaments, where negative self-schemas were predominant, hyperthymic temperament showed nearly equivalent mediation effects for both positive and negative self-schemas, highlighting its unique psychological profile. This may reflect the temperament’s adaptive qualities (e.g., high energy, optimism) as theorized in Akiskal and collaborators (5), which could enhance positive self-perceptions while reducing negative ones.

In summary, beyond identifying a general therapeutic target, our findings help prioritize intervention strategies. The consistently stronger and broader mediating role of negative self-schemas suggests that cognitive therapies should prioritize the modification of these maladaptive core beliefs across all affective temperaments. In contrast, the more circumscribed role of positive self-schemas indicates that interventions aimed at bolstering positive self-views—while valuable—may be most efficaciously deployed as an adjunctive strategy, particularly for individuals with high hyperthymic traits.

### 4.3 Limitations

The present study has several limitations that should be considered. First, its cross-sectional design precludes drawing conclusions about causal inferences. Without longitudinal or experimental data, we cannot establish the temporal precedence necessary to clarify directionality. As such, the estimated indirect effects should be interpreted not as evidence of a causal mechanism, but as a pattern of association consistent with our mediation model, providing initial evidence for the potential role of self-schemas. Second, part of the participants were Psychology students, so their prior knowledge of psychological concepts may have influenced their responses, potentially introducing bias. Additionally, the proportion of women in the sample was overrepresented and participants were predominantly young adults, which limit the generalizability of the findings to broader community populations. Third, the interpretation of our findings must consider the challenge of measuring theoretically related constructs. The potential conceptual and item overlap between measures of self-schemas, affective temperaments, and affective symptoms poses a threat to discriminant validity, potentially inflating their relationships. However, temperaments and self-schemas are well-established potential risk-factors for emotional distress, and they are considered more stable and trait-like (77–79), whereas symptoms represent state-like clinical manifestations that fluctuate over time (80, 81). Fourth, although we controlled for age and gender as covariates in the mediation analyses, we did not formally test whether the observed pathways were moderated by gender or other demographics. Future research with more balanced samples should explicitly investigate whether the strength of the relationships between affective temperaments, self-schemas, and emotional distress varies across demographic groups. Finally, although the instruments employed to measure variables are well-validated, they are self-report assessments, which introduces the risk of common-method variance and subjective biases, such as recall inaccuracies. Future studies could benefit from multi-method assessments (e.g., ecological assessment, informant-report measures) to complement data.

### 4.4 Conclusions

This study addresses a critical gap in the literature by providing the first empirical evidence of self-schemas as mediating mechanisms linking affective temperaments to affective symptoms. The dominant role of negative self-schemas in the pathways from affective temperaments to emotional symptoms highlights their transdiagnostic nature, offering a parsimonious explanation for comorbidity. Overall, findings suggest that interventions targeting maladaptive self-schemas, particularly negative ones, could disrupt pathways from temperamental vulnerability to emotional distress, while strategies to enhance positive self-schemas could hold additional value for depressive symptoms and specific temperament profiles. Ultimately, these findings support a more integrated biopsychosocial model of emotional distress, highlighting the dynamic interplay between temperament—potentially rooted in biological predispositions—and cognitively constructed elements shaped by experience, such as self-schemas, in influencing psychological outcomes. Further longitudinal and clinical studies are needed to confirm the causal nature of these relationships and to translate these findings into effective cognitive interventions.

## Data Availability

The raw data supporting the conclusions of this article will be made available by the authors, without undue reservation.
